# Effects of Incremental Urea Supplementation on Rumen Fermentation, Nutrient Digestion, Plasma Metabolites, and Growth Performance in Fattening Lambs

**DOI:** 10.3390/ani9090652

**Published:** 2019-09-04

**Authors:** Yixuan Xu, Zhipeng Li, Luis E. Moraes, Junshi Shen, Zhongtang Yu, Weiyun Zhu

**Affiliations:** 1 Laboratory of Gastrointestinal Microbiology, Jiangsu Key Laboratory of Gastrointestinal Nutrition and Animal Health, College of Animal Science and Technology, Nanjing Agricultural University, Nanjing 210095, China (Y.X.) (Z.L.) (W.Z.); 2 National Center for International Research on Animal Gut Nutrition, Nanjing Agricultural University, Nanjing 210095, China; 3 Department of Special Animal Nutrition and Feed Science, Institute of Special Animal and Plant Sciences, Chinese Academy of Agricultural Sciences, Changchun 130112, China; 4 Department of Animal Sciences, The Ohio State University, Columbus, OH 43210, USA (L.E.M.) (Z.Y.)

**Keywords:** urea, fattening Hu lamb, growth performance, nutrient digestion, rumen fermentation

## Abstract

**Simple Summary:**

Soybean meal is a major protein ingredient in ruminant diets. However, the swine and poultry industries are also competitors for soybean meal as their primary protein ingredient. Thus, soybean meal is expensive, and actually the most expensive gradient of ruminant diets. In this context, urea is used as a low-cost nitrogen source to replace up to 75% of the soybean meal typically fed to fattening lambs. Urea at 10 g could substitute 130 g soybean meal per kg feed dry matter without adverse effects on digestion, metabolism, or growth in fattening lambs when fed a high concentrate diet.

**Abstract:**

This study investigated the effects of partially substituting soybean meal (SBM) with incremental amount of urea on rumen fermentation, nutrient digestion, plasma metabolites, and growth performance in fattening lambs. Seventy fattening male lambs were sorted into two blocks according to body weight (BW) and assigned to one of five dietary treatments in a randomized block design: SBM at 170 g/kg dry matter (DM) or reduced SBM (40 g/kg DM) plus 0, 10, 20, or 30 g urea/kg DM. Compared with the lambs receiving the SBM diet, the lambs fed the reduced SBM diet plus urea had higher (*p* < 0.01) concentrations of ruminal ammonia, and the ruminal concentration of ammonia also increased linearly (*p* < 0.01) with the increasing urea supplementation. Linear and quadratic effects (*p* < 0.01) on the crude protein (CP) intake and digestibility were observed with the increasing urea addition to the diet. The concentrations of plasma ammonia and blood urea nitrogen (BUN) increased (linear, *p* < 0.01; quadratic, *p* < 0.01) with the increasing urea supplementation. The final BW, DM intake (DMI), average daily gain (ADG), and gain efficiency were similar (*p* ≥ 0.42) between the SBM group and the urea-supplemented groups. However, the DMI and ADG increased quadratically (*p* ≤ 0.03) with the increasing urea addition to the diet, with the 10 g urea/kg DM diet resulting in the highest DMI and ADG. The results of this study demonstrated that 10 g urea could substitute 130 g soybean meal per kg feed DM without any adverse effect on growth performance or health in fattening lambs when fed a high concentrate diet.

## 1. Introduction

Ruminant animals, such as cattle, sheep, and goats, have a unique ability to convert plant protein, especially poor-quality protein, and non-protein nitrogen, including urea, into high-quality animal proteins as meat or milk. This ability is attributed to the rumen microbiome. In the current intensive ruminant production, however, most producers use a high-concentrate diet to improve animal production performance [1]. Soybean meal is the preferred and major protein ingredient in ruminant diets, but soybean is an important grain for human consumption, especially in Asian and other developing countries. The swine and poultry industries are also competitors for soybean as their primary protein ingredient. Thus, soybean is high-priced, actually the most expensive gradient of all animal diets. Moreover, soured international trading relationship, as illustrated by the recent USA-China trade spat, can further raise the price of soybean and increase the cost of animal feeding operations in some countries. Therefore, researchers have been searching for alternative nitrogen sources, both plant-based protein and non-protein nitrogen, for ruminant production.

Urea is a non-protein nitrogen feedstuff high in nitrogen content but low in price. Since the mid-1950s, researchers have explored urea as a low-cost nitrogen source to replace a portion of the protein ingredient in ruminant diets [2]. Numerous studies have shown that when fed at appropriate levels, urea had no adverse influence on the rumen fermentation [3], dry matter intake (DMI) [4,5], nutrition digestibility [6], or growth performance [7] in ruminants. However, Wang et al. [8] reported that DMI and weight gain were reduced in Dorper crossbred sheep when urea was supplemented at up to 25 g/kg dry matter (DM). It is assumed that when the amount of supplemental urea exceeds the maximum concentration that inhibits the rumen microbiome, it can decrease animal performance and even cause ammonia toxicity [9].

Feeding of high-concentrate diet increases the production of volatile fatty acids (VFAs) in the rumen [10] and decreases the ruminal pH [11]. Abdoun et al. [12] reported that when the physiological pH of rumen liquid is 6.5 or lower, the ruminal ammonia is primarily absorbed in the form of NH_4_^+^. The NH_4_^+^ absorption across the rumen epithelium requires the assistance of carriers or channels, and it is slower compared to that of NH_3_ [13]. Theoretically, low rumen pH can decrease the absorption rate of ammonia from the rumen into the blood stream, lowering the risk of ammonia toxicity. Besides, the increased microbial growth in high energetic diets may also partially alleviate the ammonia toxicity. Therefore, we hypothesized that urea, when fed together with a high-concentrate diet, may partially substitute dietary protein without decreasing animal growth performance or increasing the risk of ammonia toxicity in ruminants. We tested this hypothesis in the present study by evaluating the effects of incremental urea supplementation on rumen fermentation, nutrient digestibility, plasma metabolites, and growth performance using fattening Hu lambs as a model.

## 2. Materials and Methods

### 2.1. Animals, Diets, and Experimental Design

The experimental procedures used in this study were approved by the Animal Care and Use Committee of Nanjing Agricultural University (protocol number: SYXK2017-0007).

Seventy male fattening Hu lambs (24.3 ± 1.7 kg initial body weight (BW)) at the age of 3 to 4 months were weighted using a scale with a precision of 100 g (Rongcheng Electronic Scale, Zhejiang, China) on 2 consecutive days at the start of the experiment and blocked by BW into two blocks (low: *n* = 40, 23.13 ± 0.7 kg initial BW; high: *n* = 30, 25.92 ± 1.3 kg initial BW). Lambs within each block were then allotted to 20 and 15 pens respectively (two lambs/pen). Pens within each block were randomly assigned to one of five dietary treatments: Soybean meal (SBM), urea supplemented at 0 g/kg (U0), urea supplemented at 10 g/kg (U10), urea supplemented at 20 g/kg (U20), and urea supplemented at 30 g/kg (U30), respectively. The SBM group received 170 g SBM per kg DM (the typical SBM inclusion level), while the U0, U10, U20, and U30 groups received much more decreased SBM (about 40 g/kg DM) plus 0, 10, 20, and 30 g, respectively, of granular urea (Jiangsu Kelunduo Food Ingredients Co. LTD., Jiangsu, China) per kg DM. All diets had similar energy content but different crude protein (CP) content (Table 1) and were formulated to meet the energy requirement of meat-producing sheep weighing 25 kg, with an assumed average daily gain (ADG) of 200 g [14]. The CP level of the SBM and U20 diets met the CP requirement, but that of the U0 and U10 diets were below while that of the U30 diet was above the CP requirement of meat-producing sheep. Feeding trials were conducted for 9 weeks, consisting of 1 week for adaptation followed by 8 weeks of dietary treatments. In the one-week adaption period, all lambs were fed the U0 diet. Then, the lambs in the SBM, U10, U20, and U30 groups were allowed to gradually transition to their diets using four step-up regimens, with each step-up being two days. All the lambs were fed a TMR having a 55:45 concentrate-roughage ratio (concentrate containing corn grain, soybean meal, wheat bran, premix and urea, and roughage including corn silage and peanut vine) twice daily at 0700 and 1900 h, and there was approximately 10% feed refusal. All the pens (4 m × 4 m) were housed indoors with wooden slatted floors and had free access to drinking water.

### 2.2. Sampling and Measurement

Rumen fluid was collected using an oral stomach tube approximately 3 h after morning feeding on the seventh day of weeks 4 and 8. The first 80 mL of the rumen sample was discarded to minimize contamination by saliva. The pH of each ruminal fluid sample was measured immediately with a portable pH meter. One mL of each ruminal fluid sample was preserved by adding 0.2 mL of 25% HPO_3_ for VFA analysis using gas chromatography (7890A, Agilent, Santa Clara, CA, USA) according to the method described by Mao et al. [15]. Another 1 mL of each ruminal fluid sample was stored at −20 °C for subsequent analysis for ammonia using a colorimetric method [16].

Diet and feed refusals samples were collected daily for 5 consecutive days (from the third to the seventh day) on weeks 2, 4, 6, and 8. Fecal samples were collected twice daily from each lamb before each feeding for 4 consecutive days (from the fourth to the seventh day) of weeks 4 and 8. Daily feed and orts were composited for each lamb for the whole experimental period, subsampled, and then stored at −20 °C until analysis. Fecal samples were composited for each pen for week 4 and 8 separately and subsequently stored at −20 °C until analysis. At the end of the experiment, all samples were thawed and dried at 65 °C for 48 h. Dried samples were ground through a 1-mm sieve using a Cyclotec mill (Tecator 1093; Tecator AB, Höganäs, Sweden) before analysis. All samples were analyzed for DM, organic matter (OM), and CP [17]. Contents of acid detergent fiber (ADF) and neutral detergent fiber (NDF) were determined according to Van Soest et al. [18]. Acid-insoluble ash (AIA) in both the diet and the fecal samples was analyzed using the method described by Van Keulen and Young [19], and the results were used as internal markers for estimating apparent total tract nutrient digestibility.

Blood samples (9 mL each) were drawn from the jugular vein of each lamb into heparinized evacuated tubes at approximately 3 h after morning feeding on the sixth day of week 4 and 8. The sample was then centrifuged at 3000× *g* for 15 min to obtain the plasma. All plasma samples were stored at −20 °C for later analysis of plasma ammonia, blood urea nitrogen (BUN), glucose, total protein, albumin, creatinine, uric acid, total cholesterol, triglyceride, alanine aminotransferase (ALT), aspartate aminotransferase (AST), and alkaline phosphatase (ALP) using respective commercial kits (Jiancheng Bioengineering Institute, Nanjing, China). Globulin was estimated as the difference between total protein and albumin.

The diet offered and orts were weighed for 5 consecutive days (from the third to the seventh day) in weeks 2, 4, 6, and 8 to determine DMI. The lambs were weighed on 2 consecutive days in weeks 4 and 8 at 06:00 a.m. before feeding. Data were used to calculate ADG (mean ADG for weeks 1 to 4 and for weeks 5 to 8). Lamb gain efficiency was calculated as the ratio of ADG to DMI.

### 2.3. Statistical Analyses

The experiment was analyzed with a randomized block design, with repeated measures in time and design structure. The experimental unit was pen, but the observational unit was either pen (when measurements or analyses were done for each pen, such as DMI) or sheep (when measurements or analyses were done on each lamb within each pen, such as blood metabolites). The statistical model included the fixed effects of treatment and week, as well as their interaction, and the random effects of block and pen × block × treatment and lambs within the pen × block × treatment. When the pen was the observational unit, the random effects in the model were reduced to block and pen × block × treatment. If no treatment × week interactions were observed for any variables, averages across weeks were presented. The correlation on the errors due to the repeated measures was modeled with a first-order autoregressive correlation structure. Treatment effects were tested using the following contrasts: (1) SBM vs. U0 and (2) SBM vs. the average of all diets containing urea (U10, U20, and U30) and (3) linear, (4) quadratic, and (5) cubic effects of U0, U10, U20, and U30 diets. Statistical analyses were performed using PROC MIXED with SAS 9.4 (SAS Institute Inc., Cary, NC, USA), and differences were considered to be statistically significant when the *p*-values were ≤0.05. Trends were declared at 0.05 < *p* ≤ 0.10. Residual analysis was used to determine if a transformation of variable was needed. If needed, reciprocal transformations were performed. All reported values are least squares means unless otherwise stated.

## 3. Results

### 3.1. Rumen Fermentation Characteristics

The effects of urea supplementation on rumen fermentation characteristics in fattening lambs are presented in Table 2. A treatment × week interaction (*p* < 0.05) was observed for pH, ammonia and acetate, but not for other rumen fermentation parameters (*p* ≥ 0.07). Compared with the lambs fed SBM diet, the ammonia concentration of lambs fed the U0 diet tended to be lower (*p* = 0.08) at week 4, but was significantly lower (*p* < 0.01) at week 8. However, the lambs fed the decreased SBM diet plus urea had a higher (*p* < 0.01) ammonia concentration than those fed SBM diet at both week 4 and 8. Moreover, the concentration of ruminal ammonia increased linearly (*p* < 0.01) with the increase of urea supplementation. No difference (*p* ≥ 0.31) in ruminal pH was noted among the dietary treatments at week 4, but the ruminal pH increased (linear, *p* < 0.01; cubic, *p* = 0.03) with increasing urea supplementation at week 8. No difference (*p* ≥ 0.06) was detected in ruminal concentrations of acetate, propionate or total VFAs among the dietary treatment groups. However, the concentrations of butyrate and isobutyrate were higher (*p* ≤ 0.01) for the lambs consuming the SBM diet compared with the lambs fed the decreased SBM diet containing urea.

### 3.2. Nutrients Intake and Apparent Digestibility

A significant interaction (*p* < 0.05) of treatment × week was found for total tract apparent digestibility of DM, OM, CP, NDF, and ether extract (EE), but not for nutrients intake (*p* > 0.20) (Table 3). The intake of DM, OM, CP, and ADF for the lambs receiving the SBM diet was higher (*p* ≤ 0.04) than the lambs receiving the U0 diet, but similar (*p* ≥ 0.62) to the lambs receiving reduced SBM plus urea. In addition, linear effect (*p* < 0.01) on the CP intake and quadratic effects (*p* ≤ 0.05) on the intake of DM, OM, NDF, and ADF were observed with the increased urea supplementation. The DM, OM, NDF, and ADF intake of lambs fed the U10 diet were numerically the highest, whereas the lambs fed U0 diet were numerically the lowest. The lambs fed the SBM diets had higher (*p* < 0.01) digestibility of ADF than those fed U0 diet, and the digestibility of ADF increased linearly (*p* = 0.02) as urea supplementation increased. The lambs fed the SBM diet had a lower (*p* < 0.01) DM digestibility than those fed U0 diet at week 8, but no difference (*p* = 0.86) was found at week 4. In contrast, the lambs fed the SBM diet had a higher (*p* ≤ 0.03) NDF and EE digestibility than those fed U0 diet at week 4, but no difference (*p* ≥ 0.17) was found at week 8. The OM and CP digestibility of the lambs consuming SBM diet were higher (*p* ≤ 0.04) than the lambs consuming U0 diet at both week 4 and 8. However, the digestibility of DM and NDF of the lambs consuming SBM diet were similar (*p* ≥ 0.18) to the lambs fed reduced SBM plus urea diet at both week 4 and 8. In addition, the digestibility of CP increased linearly (*p* < 0.01) with increasing urea supplementation at both week 4 and 8.

### 3.3. Plasma Metabolites

The data of plasma metabolites are presented in Table 4. A treatment × week interaction (*p* < 0.05) was observed for BUN, glucose, globulin, and triglyceride, but not for other plasma metabolites (*p* ≥ 0.06). The concentration of plasma ammonia for the lambs fed the SBM diet were higher (*p* < 0.01) than the lambs fed the U0 diet, but lower (*p* = 0.03) than the lambs fed the decreased SBM diet containing urea. The concentration of BUN for the SBM group was higher (*p* < 0.01) than the U0 group at week 4 and 8. However, compared with the lambs fed the SBM diet, the lambs fed the reduced SBM diet plus urea had similar (*p* = 0.62) BUN concentration at week 4, but higher (*p* < 0.01) BUN concentration at week 8. In addition, the concentrations of plasma ammonia and BUN increased (linear, *p* < 0.01; quadratic, *p* < 0.01) as the percentage of urea incrementally increased in the diet at both weeks. The globulin concentration decreased linearly (*p* < 0.01) and quadratically (*p* = 0.02) as increasing urea in the diet at week 4, but only a linear decrease (*p* < 0.01) was found at week 8. The lambs fed the SBM diets had lower (*p* ≤ 0.01) globulin concentration than those fed the U0 diet at both weeks. The lambs fed the SBM diet had higher (*p* ≤ 0.04) glucose concentration than those fed the U0 or reduced SBM plus urea diet at week 8, and no difference (*p* ≥ 0.10) was found at week 4. In contrast, the lambs fed the SBM diet had lower (*p* < 0.01) triglyceride concentration than those the fed U0 or reduced SBM plus urea diet at week 4, and no difference (*p* ≥ 0.22) was found at week 8. The concentrations of total protein, creatinine, and total cholesterol in the lambs fed the SBM diet were lower (*p* ≤ 0.04) than in the lambs fed the U0 diet, but similar (*p* ≥ 0.07) to the lambs fed urea at the tested concentrations. Moreover, no difference (*p* ≥ 0.15) in levels of uric acid, ALP, AST, and ALT were noted between the SBM group and U0 group or urea-supplemented groups.

### 3.4. Growth Performance

No interaction (*p* ≥ 0.19) of treatment × week was detected on lambs growth performance (Table 5). The final BW and gain efficiency were similar (*p* ≥ 0.17) between the lambs fed the SBM diet and those fed the decreased SBM diet irrespective of urea supplementation. However, the average final BW tended to increase quadratically (*p* = 0.06) with increasing urea supplementation in the diet. The final BW of the lambs receiving the U10 diet was highest, about 2 kg heavier than that of the lambs receiving the U0 diet. The lambs consuming the SBM diet achieved a greater DMI and ADG (*p* ≤ 0.03) than the lambs consuming the reduced SBM diet, but not than the lambs consuming the reduced SBM diet plus urea (*p* ≥ 0.70). In addition, quadratic effect (*p* ≤ 0.03) on DMI and ADG were observed with the increasing urea supplementation. The DMI and ADG of lambs fed the U10 diet were numerically the highest, whereas the lambs fed U0 diet were numerically the lowest.

## 4. Discussion

### 4.1. Rumen Fermentation Characteristics

Ammonia is an important source of nitrogen for microbial protein synthesis and growth in the rumen [12]. The most suitable concentration of rumen ammonia is about 8.8 mg/dL [20]. However, ruminants can suffer from ammonia toxicity when rumen ammonia concentration exceeds 140 mg/dL [21]. Rumen ammonia concentration is determined by both the nitrogen level of diet and the ruminal urea influx via the urea circulation. Patra et al. [9] reported that ruminal ammonia concentration is the major negative regulator of urea influx. Urea, which can be rapidly hydrolyzed to ammonia by rumen ureolytic microbes (primarily bacteria), is 100% ruminally degradable [22]. Moreover, the amount of urea added to diets often far exceeded the amount of ruminal urea influx. Therefore, the rumen concentration of ammonia expectedly increased linearly with increasing urea supplementation in diet, corroborating the results of Spanghero et al. [7], who showed that accumulation of ammonia was positively correlated with the quantity of degradable CP. However, the ruminal ammonia concentration observed in the lambs fed the U30 diet was far below the toxic level reported by Lewis et al. [21]. Moreover, the ruminal pH values of all the treatments were within the normal physiological range, 6.1 to 6.8 [18]. The ruminal pH is an indication of the balance between the level of ammonia and total VFAs in the rumen. The similar total VFAs and the linearly increasing ruminal ammonia concentration with the increase of urea supplementation explain the linearly increased rumen pH with increasing urea supplementation in the diet.

In the current study, we found that the concentrations of acetate, propionate, and total VFAs were not influenced by the urea supplementation, which is consistent with the research of Currier et al. [3]. The ruminal VFAs are derived mainly from the microbial fermentation of dietary carbohydrates. Therefore, the similar proportion of carbohydrates in the study diets is expected to lead to similar concentrations of acetate, propionate, or total VFAs across all the treatments. However, the concentration of total VFAs in the lambs fed the U0 diet was numerically the lowest compared to the other groups. Given the low ruminal ammonia concentration in the lambs fed the U0 diet, which is far below 8.4 mg/dL that was thought to be optimal for microbial growth [23], the rumen fermentation and VFAs production in the lambs fed the U0 diet might have been suppressed to some extent. Additionally, we also found that the lambs feeding the SBM diet had a higher rumen concentration of isobutyrate than those fed decreased SBM plus urea, which agrees with several previous studies [24]. This might have probably resulted from deamination of the branched-chain amino acids present in soybean meal [24]. These results suggest that substitution of SBM with urea may affect rumen fermentation to some extent but not necessarily lower the growth of fattening sheep when they are fed a high-concentrate diet.

### 4.2. Feed Intake and Apparent Digestibility

Many researchers have reported the influence of adding urea on feed intake and apparent digestibility in ruminants. Sweeny et al. [5] observed that Merino sheep fed oaten chaff hay treated with urea had a greater average DMI compared to sheep receiving untreated oaten chaff hay. In contrast, Spanghero et al. [7] reported that when substituting SBM with 5 g urea per kg DM, no difference in DM and OM intake or digestibility was observed. Additionally, Wang et al. [8] reported that when urea was supplemented at up to 25 g/kg DM, the DMI was reduced in Dorper crossbred sheep. The conflicting results were probably related to the variation of experimental animals and diets. Increased hay intake and digestibility in response to urea supplementation could be attributed to the synchronization of carbohydrate and nitrogen utilization in the rumen, which could increase the rate of microbial growth and fermentation therein. In this research, the intakes of DM, OM, CP, and ADF were higher for the lambs receiving the SBM diet than those receiving the U0 diet, but similar to the lambs receiving reduced SBM plus urea. This might be the result of the improvement of microbial fermentation when ammonia concentration increased in the rumen. Contradicting to the finding of Wang [8], supplementation of urea at 30 g/kg DM did not significantly lower feed intake or digestibility, which might be attributed to the high-concentrate diet used in the current study. As discussed above, high concentrate diet increases total VFAs production and decrease the pH in the rumen, which can slow the absorption of rumen ammonia into the blood [12], lowering ammonia toxicity in the lambs. Additionally, according to the calculation formula of CP apparent digestibility, the apparent digestibility of CP is positively correlated with dietary CP level when the metabolic fecal nitrogen is constant [25]. Therefore, the lambs consuming the SBM diet were higher than those fed the U0 diet and there was a linear increase with incremental urea supplementation in CP apparent digestibility. These results indicate that feed intake and digestibility can be maintained when urea partially substitute SBM in ruminant diets.

### 4.3. Plasma Metabolites

Plasma concentrations of blood metabolites collectively represent an integrated index of the utilization of nutrients and indicate nutritional status [26]. In the present study, the levels of plasma metabolites were within or close to the normal range for healthy sheep [5,27], which suggests that all the treatments did not adversely impact these metabolite profiles. As expected and shown in previous study, plasma ammonia and urea concentrations were elevated with the increased supplementation [28]. Ammonia can cause adverse effects ranging from depressed animal performance to death [9]. The highest level of blood ammonia (226.9 μg/dL noted in the U30 group) was still far below the toxicity level of 800 μg/dL reported by Webb et al. [29]. Furthermore, the liver is the most important organ that converts ammonia into urea as a non-toxic end product, which is excreted via the kidney, and the activities of plasma ALP, AST, and ALT are common indicators of hepatic injury. The normal and similar levels of ALP, AST, and ALT activities among all groups, including those fed urea at 30 g/kg DM indicate that the urea supplementation did not impair liver function or raise the risk of ammonia toxicity.

Blood glucose concentration in ruminants is partially affected by gluconeogenesis, and precursor availability is an important factor in regulating gluconeogenesis [30]. In ruminants, propionate is the only gluconeogenic VFA, and the similar ruminal propionate concentrations in the SBM and urea-supplemented groups are consistent with the similar plasma glucose concentrations detected in those groups, a finding corroborating those of several previous studies [27]. The levels of blood globulin and total protein are related to the humoral immunity and protein synthesis of animals [31]. The higher concentrations of globulin and total protein in the lambs fed the U0 diet than in those fed the SBM diet might be attributed to the low level of CP in the U0 diet, but the specific mechanism remains to be elucidated. Nevertheless, partial substitution of SBM with 10 g urea per kg DM probably will not adversely affect the metabolism of lambs.

### 4.4. Growth Performance

When lambs are raised with under health conditions, their BW gain primarily depends on the DMI and digestibility [32]. Consistent with the similar DMI and digestibility between the lambs fed the SBM diet and those fed the decreased SBM diet plus urea, their final BW were also no different. However, the final BW of the lambs receiving the U10 diet was the highest, and about 2 kg heavier than that of the lambs receiving the U0 diet. The improved animal growth of the U10 group could be attributed to the synchronization of carbohydrate and nitrogen availability in the rumen. In one study, Wang et al. [8] found that urea supplementation at up to 15 g/kg DM did not influence BW gain, but urea supplementation at 25 g/kg DM reduced the growth performance of Dorper crossbred sheep fed a diet with a 50:50 concentrate:forage ratio. The decreased animal growth might be explained as a result of disruption of the synchronization between carbohydrate and nitrogen due to a rapid increase in ammonia concentration in the rumen [33]. Contradicting to the study of Wang et al. [8], however, the present study showed no negative influence on lamb growth performance when urea was fed at 30 g/kg DM. This discrepancy is probably due to the difference in the composition of the diets and/or lamb breed. Thus, the optimal level of urea supplementation needs to be determined for the specific diet fed to the animals and breed of interest. Furthermore, because urea supplementation can increase nitrogen excretion, the potential pollution of the environment with nitrogen should be considered when the level of urea supplementation is determined.

## 5. Conclusions

Urea can partially substitute soybean meal to raise fattening lambs when fed concentrate-based diets without impairing nutrients utilization, ruminal fermentation, metabolism, or growth performance. Concentrate-based diet may improve the synchronization between feed fermentation/VFA production and ammonia assimilation. Urea at 10 g/kg DM feed may substitute 75% of the soybean meal typically fed (170 g/kg DM feed) to fattening lambs.

## Figures and Tables

**Table 1:** Ingredient and chemical composition of the experimental diets.

Item	Dietary Treatments				
	SBM	U0	U10	U20	U30
Ingredient, g/kg DM					
Corn silage	250.0	250.0	247.5	245.1	242.7
Peanut vine	200.0	200.0	198.0	196.1	194.2
Corn grain	290.0	420.0	415.8	411.8	407.8
Soybean meal	170.0	40.0	39.6	39.2	38.8
Wheat bran	40.0	40.0	39.6	39.2	38.8
Premix ^1^	50.0	50.0	49.5	49.0	48.5
Urea	0.0	0.0	10.0	20.0	30.0
Nutrient composition, g/kg DM					
CP	175.5	115.9	144.9	172.9	200.6
NDF	316.0	326.7	331.2	319.4	328.3
ADF	207.1	203.9	213.3	208.0	208.5
EE	28.9	30.8	31.2	30.8	31.7
Ash	90.0	91.2	91.2	91.1	90.9
DE, MJ/kg [14]	13.6	13.8	13.6	13.5	13.4

SBM, soybean meal; U0, urea supplemented at 0 g/kg dry matter (DM); U10, urea supplemented at 10 g/kg DM; U20, urea supplemented at 20 g/kg DM; U30, urea supplemented at 30 g/kg DM; DM, dry matter; CP, crude protein; NDF, neutral detergent fiber; ADF, acid detergent fiber; EE, ether extract; DE, digestive energy. ^1^ Formulated to provide (per kg of DM): 150 g of salt, 200 g of NaHCO_3_, 75 g of Ca, 20 g of P, 600 mg of Mn, 680 mg of Fe, 960 mg of Zn, 300 mg of Cu, 140,000 IU of vitamin A, 55,000 of vitamin D3, 700 IU of vitamin E, and 600 mg of niacin.

**Table 2:** Effects of urea supplementation on rumen fermentation characteristics in fattening lambs.

Item	SBM	Urea Supplementation, g/kg DM				SEM	Contrast ^1^ *p*-Value				
		U0	U10	U20	U30		A	B	Linear	Quadratic	Cubic
pH ^2^											
Week 4	6.42	6.43	6.38	6.43	6.47	0.072	0.90	0.91	0.56	0.49	0.73
Week 8	6.37	6.27	6.18	6.51	6.60	0.072	0.31	0.44	<0.01	0.18	0.03
Ammonia ^2^, mg/dL											
Week 4	8.72	4.69	8.33	14.31	32.67	2.073	0.08	<0.01	<0.01	<0.01	0.16
Week 8	10.69	4.06	16.65	27.11	34.87	2.073	<0.01	<0.01	<0.01	0.13	0.94
Total VFAs, m*M*	113.6	105.7	110.5	107.0	111.9	2.81	0.06	0.26	0.25	0.99	0.21
Acetate ^2^, m*M*											
Week 4	74.3	68.6	68.9	71.5	79.5	2.38	0.10	0.73	<0.01	0.11	0.78
Week 8	70.1	65.4	72.5	65.6	67.6	2.38	0.17	0.57	0.98	0.30	0.04
Propionat, m*M*	24.7	25.1	26.2	24.5	23.5	1.10	0.81	0.98	0.19	0.35	0.46
Acetate: Propionate	3.02	2.79	2.77	2.88	3.24	0.107	0.13	0.67	<0.01	0.08	0.80
Butyrate, m*M*	13.2	10.7	10.9	11.0	11.7	0.42	<0.01	<0.01	0.08	0.56	0.77
Valerate, m*M*	1.30	1.00	1.11	1.12	1.20	0.055	<0.01	0.03	0.03	0.76	0.51
Isobutyrat, m*M*	0.94	0.75	0.69	0.75	0.78	0.041	<0.01	<0.01	0.36	0.23	0.34
Isovalerate, m*M*	1.29	1.17	0.98	1.13	1.13	0.094	0.40	0.07	0.96	0.33	0.25

SBM, soybean meal; U0, urea supplemented at 0 g/kg DM; U10, urea supplemented at 10 g/kg DM; U20, urea supplemented at 20 g/kg DM; U30, urea supplemented at 30 g/kg DM; SEM, standard error of means; VFAs, volatile fatty acids. ^1^ Contrast A = SBM vs. U0; Contrast B = SBM vs. the average of all diets containing urea (U10, U20, and U30); Contrast linear, quadratic, and cubic = Linear, quadratic, and cubic contrasts of U0, U10, U20, and U30 diets. ^2^ *p* < 0.05 for the effect of treatment × week.

**Table 3:** Effects of urea supplementation on feed intake and apparent total tract digestibility of nutrients in fattening lambs.

Item	SBM	Urea Supplementation, g/kg DM				SEM	Contrast ^1^ *p*-Value				
		U0	U10	U20	U30		A	B	Liner	Quadratic	Cubic
Intake, g/d											
DM	999.3	893.5	1011.2	1008.1	972.5	58.24	0.04	0.97	0.15	0.04	0.57
OM	909.4	812.0	919.7	916.2	884.1	52.95	0.04	0.94	0.15	0.04	0.57
CP	175.2	103.8	146.7	174.2	194.8	9.05	<0.01	0.62	<0.01	0.06	0.74
NDF	315.8	291.9	335.1	322.0	319.2	18.86	0.15	0.47	0.19	0.05	0.20
ADF	207.0	182.2	215.8	209.7	202.8	12.11	0.02	0.78	0.10	<0.01	0.25
EE	28.9	27.5	31.6	31.0	30.8	1.78	0.38	0.08	0.06	0.06	0.32
Digestibility, g/kg											
DM ^2^											
Week 4	719.5	717.5	719.3	729.8	734.1	7.67	0.86	0.36	0.086	0.87	0.67
Week 8	707.6	737.6	723.6	725.5	708.8	7.67	<0.01	0.19	0.02	0.86	0.32
OM ^2^											
Week 4	803.9	756.9	771.2	781.7	791.3	6.49	<0.01	<0.01	<0.01	0.72	0.92
Week 8	794.3	775.3	775.7	777.5	769.2	6.49	0.04	<0.01	0.57	0.50	0.70
CP ^2^											
Week 4	800.3	655.1	749.7	784.1	809.2	7.96	<0.01	0.04	<0.01	<0.01	0.16
Week 8	793.3	688.1	762.8	789.9	790.5	7.96	<0.01	0.19	<0.01	<0.01	0.19
NDF ^2^											
Week 4	580.8	528.0	544.0	563.8	593.8	16.35	0.02	0.47	<0.01	0.66	0.93
Week 8	570.2	553.1	548.4	543.1	543.4	16.35	0.46	0.18	0.64	0.88	0.93
ADF	597.8	543.7	564.5	574.4	591.3	13.56	<0.01	0.19	0.02	0.88	0.77
EE ^2^											
Week 4	897.5	865.6	877.2	884.4	885.6	5.84	<0.01	0.03	0.01	0.38	0.95
Week 8	883.4	882.8	879.9	873.9	868.0	5.84	0.94	0.17	0.06	0.80	0.90

SBM, soybean meal; U0, urea supplemented at 0 g/kg DM; U10, urea supplemented at 10 g/kg DM; U20, urea supplemented at 20 g/kg DM; U30, urea supplemented at 30 g/kg DM; SEM, standard error of means; DM, dry matter; OM, organic matter; CP, crude protein; NDF, neutral detergent fiber; ADF, acid detergent fiber; EE, ether extract. ^1^ Contrast A = SBM vs. U0; Contrast B = SBM vs. the average of all diets containing urea (U10, U20, and U30); Contrast linear, quadratic, and cubic = Linear, quadratic, and cubic contrasts of U0, U10, U20, and U30 diets. ^2^ *p* < 0.05 for the effect of treatment × week.

**Table 4:** Effects of urea supplementation on plasma metabolites in fattening lambs.

Item	SBM	Urea Supplementation, g/kg DM				SEM	Contrast ^1^ *p*-Value				
		U0	U10	U20	U30		A	B	Linear	Quadratic	Cubic
Ammonia, μg/dL	158.6	125.4	135.6	163.9	226.9	10.05	<0.01	0.03	<0.01	<0.01	0.59
BUN ^2^, mg/dL											
Week 4	29.8	14.9	25.0	29.9	32.5	1.15	<0.01	0.62	<0.01	<0.01	0.56
Week 8	28.9	15.4	27.4	33.5	36.8	1.17	<0.01	<0.01	<0.01	<0.01	0.59
Glucose^2^, mg/dL											
Week 4	76.7	83.7	82.9	81.9	74.4	3.19	0.10	0.37	0.08	0.27	0.63
Week 8	87.9	78.7	78.7	76.2	76.9	3.23	0.04	<0.01	0.59	0.89	0.66
Globulin ^2^, g/dL											
Week 4	4.19	4.89	4.24	4.19	4.49	0.179	<0.01	0.35	0.02	<0.01	0.61
Week 8	3.72	4.13	4.05	3.59	3.80	0.180	0.01	0.47	<0.01	0.21	0.06
Albumin, g/dL	2.37	2.28	2.30	2.26	2.27	0.045	0.16	0.06	0.72	0.90	0.62
Total protein, g/dL	6.32	6.79	6.44	6.15	6.40	0.182	<0.01	0.94	<0.01	<0.01	0.27
Creatinine, mg/dL	0.55	0.62	0.59	0.58	0.56	0.016	<0.01	0.19	0.03	0.44	0.91
Uric acid ^3^, mg/dL	9.54	7.76	6.80	10.00	8.05	1.089	0.24	0.29	0.39	0.63	0.052
Total cholesterol, mg/dL	57.0	65.6	62.5	63.1	63.0	3.12	0.04	0.07	0.60	0.61	0.73
Triglyceride ^2^, mg/dL											
Week 4	25.3	47.0	36.1	37.0	34.8	3.14	<0.01	<0.01	0.01	0.17	0.29
Week 8	25.9	31.3	33.8	28.7	28.6	3.14	0.23	0.22	0.35	0.68	0.40
ALP, IU/L	107.2	108.8	102.8	112.4	104.6	4.56	0.81	0.89	0.88	0.85	0.10
AST, IU/L	23.6	24.6	24.7	23.3	22.1	2.02	0.73	0.89	0.30	0.75	0.84
ALT, IU/L	476.0	422.0	455.6	422.1	435.6	37.14	0.32	0.37	0.97	0.79	0.49

SBM, soybean meal; U0, urea supplemented at 0 g/kg DM; U10, urea supplemented at 10 g/kg DM; U20, urea supplemented at 20 g/kg DM; U30, urea supplemented at 30 g/kg DM; SEM, standard error of means; BUN, blood urea nitrogen; ALP, alkaline phosphatase; AST, aspartate aminotransferase; ALT, alanine aminotransferase. ^1^ Contrast A = SBM vs. U0; Contrast B = SBM vs. the average of all diets containing urea (U10, U20, and U30); Contrast linear, quadratic, and cubic = Linear, quadratic, and cubic contrasts of U0, U10, U20, and U30 diets. ^2^ *p* < 0.05 for the effect of treatment × week. ^3^ Reciprocal transformation value.

**Table 5:** Effects of urea supplementation on body weight, dry matter intake, average daily gain, and gain efficiency in fatting lambs.

Item	SBM	Urea Supplementation, g/kg DM				SEM	Contrast ^1^ *p*-Value				
		U0	U10	U20	U30		A	B	Linear	Quadratic	Cubic
BW, kg											
Initial	24.4	24.4	24.6	24.6	24.6	1.54	0.96	0.83	0.85	0.87	0.90
Final	34.9	33.9	35.8	35.3	35.2	1.54	0.17	0.43	0.17	0.06	0.23
DMI, g/d	1000.0	892.3	1023.3	999.5	976.7	53.69	0.03	0.99	0.14	0.03	0.32
ADG, g/d	190.6	167.2	199.9	191.1	189.4	6.29	0.02	0.70	0.055	0.01	0.10
Gain efficiency, kg/kg	0.192	0.179	0.197	0.192	0.196	0.006	0.18	0.66	0.13	0.29	0.28

SBM, soybean meal; U0, urea supplemented at 0 g/kg DM; U10, urea supplemented at 10 g/kg DM; U20, urea supplemented at 20 g/kg DM; U30, urea supplemented at 30 g/kg DM; SEM, standard error of means; BW, body weight; DMI, dry matter intake; ADG, average daily gain. ^1^ Contrast A = SBM vs. U0; Contrast B = SBM vs. the average of all diets containing urea (U10, U20, and U30); Contrast linear, quadratic, and cubic = Linear, quadratic, and cubic contrasts of U0, U10, U20, and U30 diets.
